# Protocol for serum exosomal miRNAs analysis in prostate cancer patients treated with radiotherapy

**DOI:** 10.1186/s12967-018-1592-6

**Published:** 2018-08-13

**Authors:** Bijaya Malla, Daniel M. Aebersold, Alan Dal Pra

**Affiliations:** 1 Department of Radiation Oncology, Inselspital, Bern University Hospital, University of Bern, Bern, Switzerland; 2 Department of Radiation Oncology, University of Miami Miller School of Medicine, Miami, FL USA

**Keywords:** Prostate cancer, Exosomes, miRNA, Biomarker, Radiation oncology

## Abstract

**Background:**

Circulating exosomes from prostate cancer (PCa) patients undergoing radiotherapy are attractive candidate biomarkers for monitoring treatment response. Multiple workflows for isolation and content characterization of exosomes in biofluids have been attempted. We report a protocol to isolate and characterize exosomal miRNAs content and assess radiation-induced changes.

**Methods:**

In this pilot study, we performed targeted exosomal miRNA profiling of 25 serum samples obtained from PCa patients with intermediate- and high-risk disease treated with curative radiotherapy (RT), and controls. Post-treatment blood samples were collected at least 28 days after radiation therapy as a paired follow-up sample. The complete workflow consisted of two phases: I) filtration and polyethylene glycol salt precipitation phase which enriched particles below 200 nm in size followed by characterization using electron microscopy, and II) flow cytometry. Finally, miRNA expression analysis between untreated and treated patient samples was performed using RNA extraction kit, and qRT-PCR.

**Results:**

In our preliminary data, 1 ml of serum from PCa patients showed higher exosomal concentration (3.68E+10) compared to controls (6.07E+08). The overall expression of exosomes after RT was found to be higher compared to untreated samples; the median value changed from 3.68E+10 to 5.40E+10; *p *= 0.52. Using electron microscopy, we were able to visualize cup-shaped vesicles with morphology and size compatible with exosomes. The bead-based flow cytometry showed positivity for exosomal tetraspanins surface markers CD63 and CD9. All five miRNAs (hsa-let-7a-5p, hsa-miR-141-3p, hsa-miR-145-5p, hsa-miR-21-5p, hsa-miR-99b-5p) have been identified in exosomes. Despite overall changes in hsa-let-7a-5p expression after radiation, the difference was significant only in the high-risk group (*p *= 0.037). In addition, the radiation response to hsa-miR-21-5p was elevated in the high-risk group compared to the intermediate group (*p *= 0.036).

**Conclusions:**

Herewith, we demonstrated a protocol for isolation of serum exosomes and exosomal miRNA amplification. The recovery of exosomal miRNAs and their differential expression after radiation treatment suggests promising biomarker potential that requires further investigation in larger patient cohorts.

**Electronic supplementary material:**

The online version of this article (10.1186/s12967-018-1592-6) contains supplementary material, which is available to authorized users.

## Background

Prostate cancer (PCa) is one of the most common malignancies in men [1]. Despite controversies in the use of prostate specific antigen (PSA) for PCa screening, it is one of the most widely utilized biomarkers, and its clinical use has an undeniable clinical importance [2]. Nonetheless, PSA alone has not provided accurate diagnostic and prognostic information. Recently, “liquid biopsies” such as circulating exosomes have gained increasing importance [3]. These vesicles not only function in removing cellular artefacts, but also play an important role in cell-to-cell communications which is due to nucleic acid, protein cargo that is deemed to reflect cell biology of originating tumor cells [4, 5]. Thus, the spectrum of exosome research is split into isolation from biofluids, functional analysis, and its potential use in clinical assays. Studies have shown that extracellular vesicles including exosomes are a better source of selective miRNAs than the whole blood [6]. The growing interest in miRNAs, a cargo component of exosomes, is due to its stability as they are either bound to specific proteins e.g. Argonaute2 protein complex (Ago2) or are contained in exosomes protecting them from lysis by RNase in blood. miRNAs (circulating and bound) have been widely investigated in PCa and have promising applicability as prognostic and/or predictive markers [7]. Cellular stress conditions with microenvironment adaptations involve the release of miRNAs, miRNAs processing as well as changes in miRNAs function [8]. In cancer, miRNAs act both as oncomirs and tumor suppressors which may equally play a role in treatment response to different stressors including radiation treatment [9, 10]. Exosomal RNAs mediate genomic instability of recipient cells indicating a stress-induced RNA cargo released due to radiation [11]. Certain miRNAs (e.g. miR-145) have been found to promote cancer proliferation and radioresistance [12, 13] which underlines the clinical potential of miRNAs in radiation oncology [14].

The technical challenges in implementing a robust method for detection of site-specific exosomes in functional studies are well-known [15]. Many techniques for exosome isolation and recovery of exosomal miRNAs have been published showing that the functional outcomes of exosomes are technique- and sample-dependent (e.g. biofluids, cell culture samples) [16]. In clinical settings, limited volume of blood may also restrict exosome concentration and miRNA profiling. Moreover, differences in sample storage, processing, RNA extraction and amplification (e.g. qRT-PCR, deep sequencing) have substantial impact on data generation and clinical applicability. miRNAs associated with PCa and radiotherapy response are summarized in our recent paper, although literature is very limited on the topic [5]. Exosomes therefore represent a promising biological marker for potential optimization of PCa radiotherapy.

## Methods

### Ethics statement and patient serum sampling

The ethical approval for this study was obtained from the Kantonale Ethikkommission Bern, Switzerland. We planned a comparative analysis of 5 candidate miRNAs at baseline [before radiation therapy (RT)] and after RT by setting up a cohort of 11 patients and 3 controls as a pilot phase from a prospective study—SAKK63/12 (http://sakk.ch/en/sakk-provides/our-trials/urogenital-tumors/sakk-6312/). Diagnosis of PCa was initially confirmed by prostate biopsy and definition of a Gleason score (Table 1). All patients were classified into intermediate risk (IR) or high-risk groups (HR) based on National Comprehensive Cancer Network (NCCN) classification [17]. The post-radiation follow-up sampling was performed at 3 months’ time interval (± 2 months) as paired samples according to SAKK 63/12 study protocols (IR_F, HR_F). Additionally, a third group included three volunteers who visited clinic for reasons unrelated to PCa (n = 3).

**Table 1:** Patient clinicopathological characteristics

Sample ID	Age	Pre-treatment PSA (ng/ml)	Pathological stages	Gleason score	Risk group based on NCCN guidelines
B15	67	5.4	cT2a cN0 cM0	3 + 4	IR
B16	76	15.8	cT2a cN0 cM0	4 + 3	IR
B17	78	6.3	cT2b cN0 cM0	3 + 4	IR
B03	82	24.4	cT1c cN0 cM0	8	HR
B60	68	17.6	cT2a cN0 M0	3 + 4	IR
C73	66	0.9	pT2a pN0 M0	3 + 4	IR
B09	71	13.30	cT3a cN0 M0	4 + 5	HR
C05	74	0.40	pT3 pN0 M0	3 + 4	HR
C12	67	0.20	pT3a pN0 M0	3 + 4	HR
B71	54	7.2	cT1c cN0 cM0	3 + 3	IR
C59	73	0.8	pT3a pN0 cM0	4 + 4	HR

*IR* intermediate risk group, *HR* high risk group, *NCCN* National Comprehensive Cancer Network

### Exosome isolation from serum

Blood was collected in S-monovette^®^ 9 ml Z-Gel blood collection tube (Sarstedt AG., Germany), kept at room temperature for half an hour followed by centrifugation at 1500*g* for 10 min to separate serum. Afterwards, the serum samples were filtered through RNA/DNA free 0.22 µm sized syringe filter and processed for exosome isolation. Commercially available polyethylene glycol (PEG) products were used for enrichment of exosomes. Total Exosome Isolation Kit (Cat. No. 4478360) from Invitrogen was used according to manufacturers’ instructions for exosome isolation from serum samples. Briefly, 1 ml of serum was added with 250 µl of isolation reagent and mixed well by gentle vortexing. The solution was incubated at 4 °C for 1 h and then centrifuged at 10,000*g* for 10 min at room temperature. The pellet was washed twice with 1 ml of PBS and discarded. The final pellet containing exosomes was re-suspended in 100 µl resuspension buffer and then stored at − 20 °C prior to RNA isolation [18].

### Nanoparticle tracking analysis

For nanoparticle tracking analysis (NTA), exosome suspension was diluted in PBS to reach the concentration range of 2 × 10^8^ − 8 × 10^8^ particles/ml as required by NanoSight NS300 (NanoSight NTA 2.3 nanoparticle tracking and analysis) [19]. Samples were introduced into the Flow-cell top plate chamber (temperature: 25 °C) and the camera level was set to obtain an image that had sufficient contrast to clearly identify particles while minimizing background noise with video recording (camera level: 10). With violet embedded laser (405 nm, max power < 70 mW) using continuous flow of sample, 360 s videos were captured for each sample.

### Transmission electron microscopy

Five microliter of exosomes suspension was added onto 200 mesh Formvar^®^ coated and glow discharged copper grids for 20 min. Excess suspension was removed with filter paper and then fixed by placing the grids on a drop of 2% paraformaldehyde for 20 min. Grids were then washed with PBS droplets for 6 times and fixed with 1% glutaraldehyde before washing them with water droplets for 6 times. The exosomes were negatively stained by placing the grids on a droplet of 4% uranyl acetate for 10 min and air dried. Samples were then examined with a transmission electron microscope (CM12, Philips, Eindhoven) equipped with a digital camera (Morada, Soft Imaging System, Münster, Germany) and image analysis software (iTEM) [20].

### Western blot analysis

We performed western blotting using primary antibodies for exosomal surface markers CD81 (SC-7637, Santa Cruz), and CD63 (1:300 dilution; SC-365604, Santa Cruz), secondary anti-mouse (1:2000 dilution; SC-2005) HRP conjugated antibodies (Fig. 3c). Briefly, 100 µl of extracted exosome suspension was mixed with RIPA buffer for 15 min on ice. The suspension was then mixed with Laemmli buffer containing 5% Beta-mercaptoethanol and denatured at 90 °C for 5 min. The protein separation was done at constant voltage of 150 V for 60 min. After blocking with 5% Bovine Serum Albumin for 1 h at room temperature, the immune-blot Polyvinylidene difluoride membrane was incubated overnight with primary antibodies at 4 °C followed by incubation with secondary antibody at 1:2000 dilution for 1 h at room temperature. Finally, SuperSignal West Pico Chemiluminescent Substrate (Thermo Scientific) was used, then exposed to X-ray film for image detection.

### Flow cytometry

We performed flow cytometry targeting the surface markers CD63, CD9 by bead capture methods using commercial kit [21]. Exosomes mixed in isolation buffer was incubated with CD63 coated Dynabeads^®^ magnetic beads (Cat. No. 10606D, Life technologies, USA) at 2–8 °C overnight. On second day, the exosomes bound to magnetic beads were stained with FITC-CD9 (Cat. No. MA1-19557) monoclonal antibody (MEM-61), and PE-CD63 (Cat. No. MA1-19650) monoclonal antibody (MEM-259) following protocol. Ten thousands events were collected using flow cytometry (BD LSR Fortessa, BD FACS Diva software). The subsequent analysis was performed on FlowJo (FlowJo Engine v3.05470).

### RNA extraction and quantification

The Total Exosome RNA and Protein Isolation kit (Cat. No. 4478545, Invitrogen) designed for isolation of small RNA from enriched exosome preparation was used following manufacturer’s recommendations. RNA concentration and quality were evaluated by Bioanalyzer 2100 Expert (Agilent Technologies, Inc.) in conjugation with Small RNA analysis kit (Cat. No. 5067-1548) using Agilent 2100 Expert software.

### TaqMan MiRNA assays

The qRT-PCR quantification was performed on total of 25 samples to determine miRNA recovery from serum exosomes. Five miRNAs were shortlisted from previously published literature that are relevant to PCa and RT (Additional file 1: Table S1) [5]. Samples were profiled using specific individual TaqMan MiRNA Assay (RNU48; Assay-ID: 001006, let-7a-5p; Assay-ID: 000377, hsa-miR-145; Assay-ID: 002278, hsa-miR-141-3p; Assay-ID: 000463, hsa-miR-21; Assay-ID: 000397, hsa-miR-99b; Assay-ID: 000436). To perform standard qRT-PCR quantification, 2 µl of RNA elution was used for reverse transcription step, while 0.66 µl of cDNA was then used for amplification cycle. Each reactions was performed in triplicates in 10 µl of reaction volume [22]. Initial data analysis was performed using the SDS v1.4 software (Applied Biosystem) supplied with the real-time 7500 Fast RealTime PCR System.

The relative expression level of miRNAs was calculated based on average threshold cycle (CT) value from three replicates. The relative quantification—also called fold change—in miRNA expression between radiated (ΔCTpost) versus baseline patient samples (ΔCTpre) was determined by using the 2^−ΔΔCT^ comparative method.

### Statistical analysis

Data show the mean of independent biological experiments with the standard error (± SE). The two-sided paired, unpaired or the one-sample t-test were used for statistical analysis and a p-value < 0.05 was deemed statistically significant. The software used for statistical analysis was R package (http://www.R-project.org).

## Results

### Exosome concentration varies after radiation exposure

**Fig. 1:**
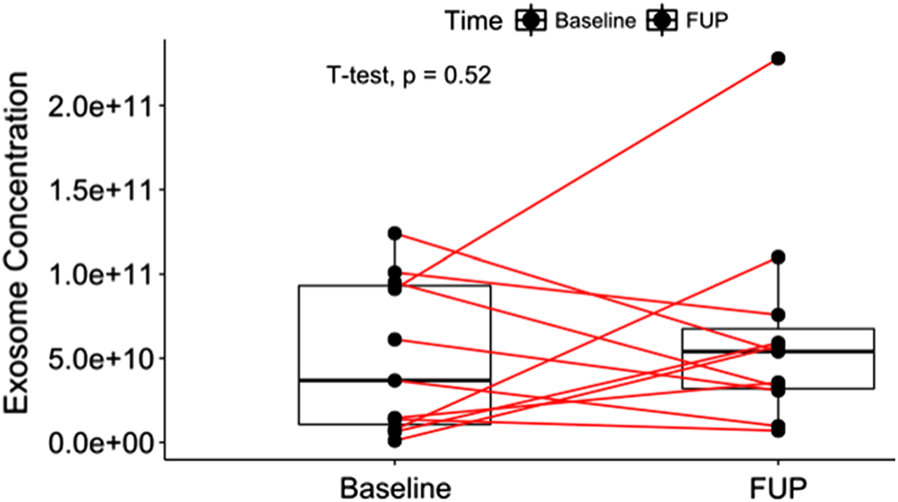
Change in longitudinal expression of exosomes in serum samples. The boxplot shows the increasing trend of exosome concentration after radiation therapy. The time difference between baseline (no radiotherapy) and FUP (radiotherapy) sampling was at least 28 days

**Fig. 2:**
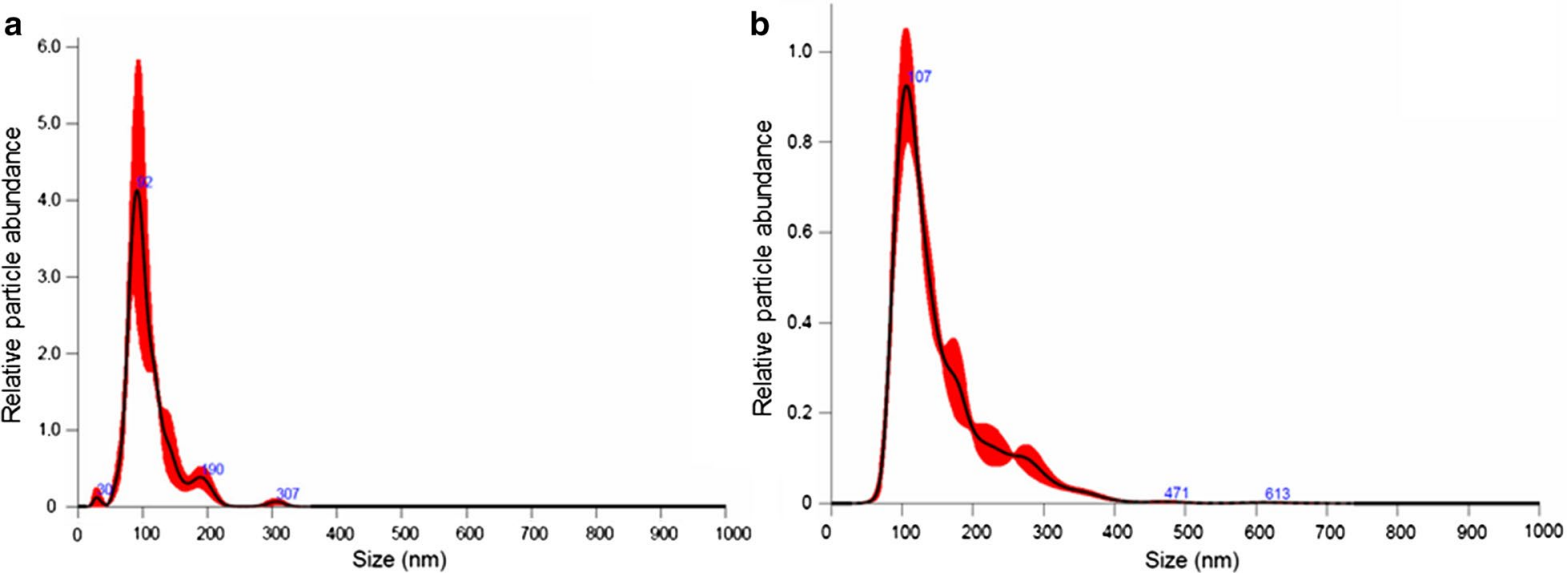
Nanosight analysis. The x-axis indicates the size distribution of particles, while y-axis shows the relative counts. The red-smear denotes variation as standard error of mean. **a** Size of most of the particles was 92 nm in baseline (no radiation) sample, **b** size of  most of the particles were 107 nm in FUP (radiation)

**Fig. 3:**
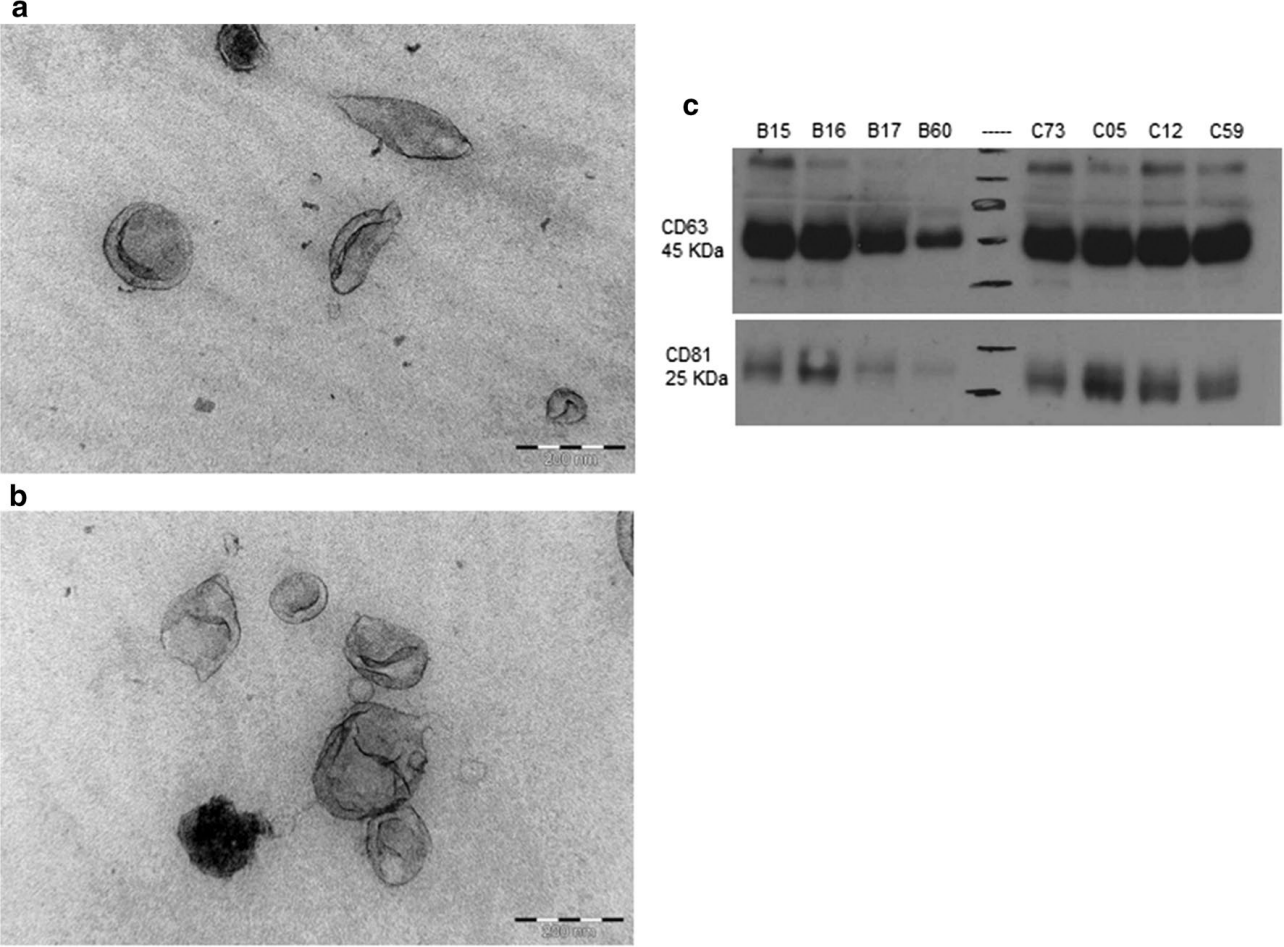
Characterization of serum derived exosomes. **a**, **b** Transmission electron microscope images of exosomes. Presence of cup-shaped vesicles sized below 200nm; **c** western blot demonstrating the expression of CD63, and CD81 in selected patients

We investigated exosome isolation workflow in 1 ml of serum using PEG based commercial kits followed by its characterization using TEM, Nanosight, and FACS [23]. We first proceeded with filtration of serum using 0.22 µm sized filter so we expected filtrate containing particles below that size [24]. To enrich the isolation of exosomes, PEG was added and the resulting pellets were suspended in buffer for further characterization steps. Using Nanosight, we observed approximately 1.89 × 10^10^ particles from 1 ml of serum from healthy controls. The overall mean concentration of vesicles were higher in PCa patients (5.02 × 10^10^ particles/ml), which increased further in post radiation serum samples (6.37 × 10^10^ particles/ml; *p *= 0.52) (Fig. 1). Majority of vesicle size obtained was between 50 and 200 nm which is considered as ideal size for exosomes (Fig. 2). The size distribution of exosomes when compared to PCa versus control, before and after radiation showed no significant difference (data not shown) which is similar to previous findings [25]. We performed western blot targeting CD63, CD81 tetraspanins surface markers known to be enriched in exosomes. The result confirmed positive bands for both proteins however the density varied across samples which demonstrates different expression of surface proteins (Fig. 3c). The western blot hybridization result were complementary to FACS result in determining the presence of exosomal surface marker protein in the sample. In TEM analysis, vesicles resembling the size of exosomes were observed as shown in Fig. 3a, b. The cup-shaped structure confirmed the morphological similarity to exosomes [18].

**Fig. 4:**
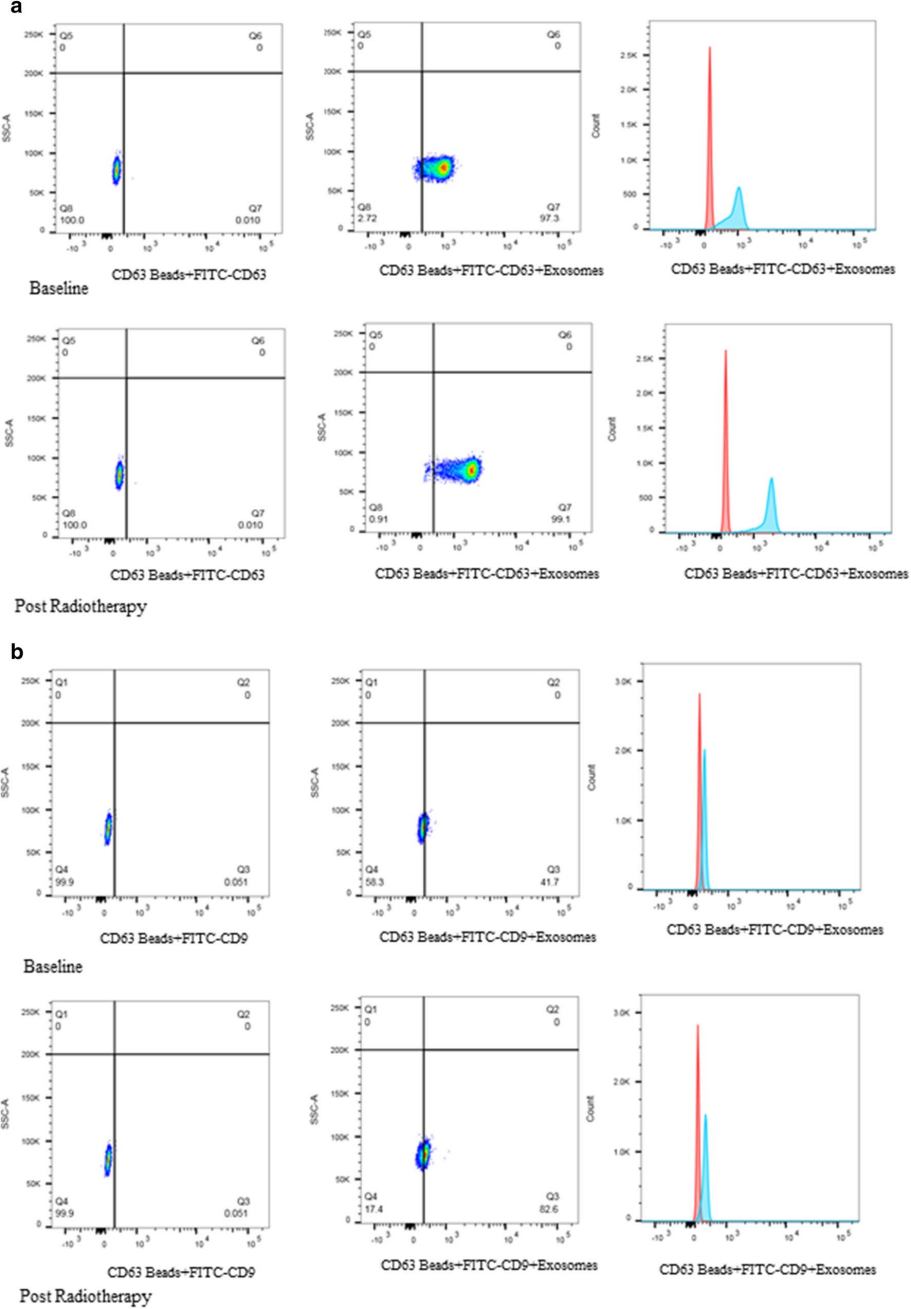
Analysis of exosomes by flow cytometry using CD63 coated magnetic beads. Exosomes were first visualized on forward (FSC) versus sidescatter (SSC) plots to gate on the respective exosomes bound to beads population, after gating on singlets. **a** Typical SSC versus PE-CD63 plots for exosomes isolated from the serum of two donor samples. As comparison, a typical plot for serum derived exosomes from prostate cancer patients using CD63 antibodies at baseline and post-radiation. **b** Typical SSC versus FITC-A plots for exosomes using CD9 antibodies at baseline and post-radiation. The graph shows increased CD63 representing exosomes compared to CD9 representing surface markers after radiotherapy

For flow cytometry analysis, CD63-beads bound exosomes were first visualized on forward (FSC) versus side scatter (SSC) graph to gate on bead singlets. Exosomes isolated using PE-CD63 antibody was flow-sorted using PE-positive gating while for those isolated with FITC-CD9 were flow-sorted using FITC-positive gating separately (Fig. 4a, b). It was distinct from data the mean fluorescent intensity for CD63 antibody was higher than for CD9 suggesting higher expression of CD63 surface marker in exosomes derived after radiation. Use of magnetic beads labelled with exosomal surface specific antibodies may increase specificity in isolating pure exosomes, however proportion of those beads may not correlate the real count of exosomes. Thus, we confined our result and discussion only as a tool to identify serum exosomes using FACS.

### Low concentration of exosomal miRNAs were characterized by qRT-PCR

**Fig. 5:**
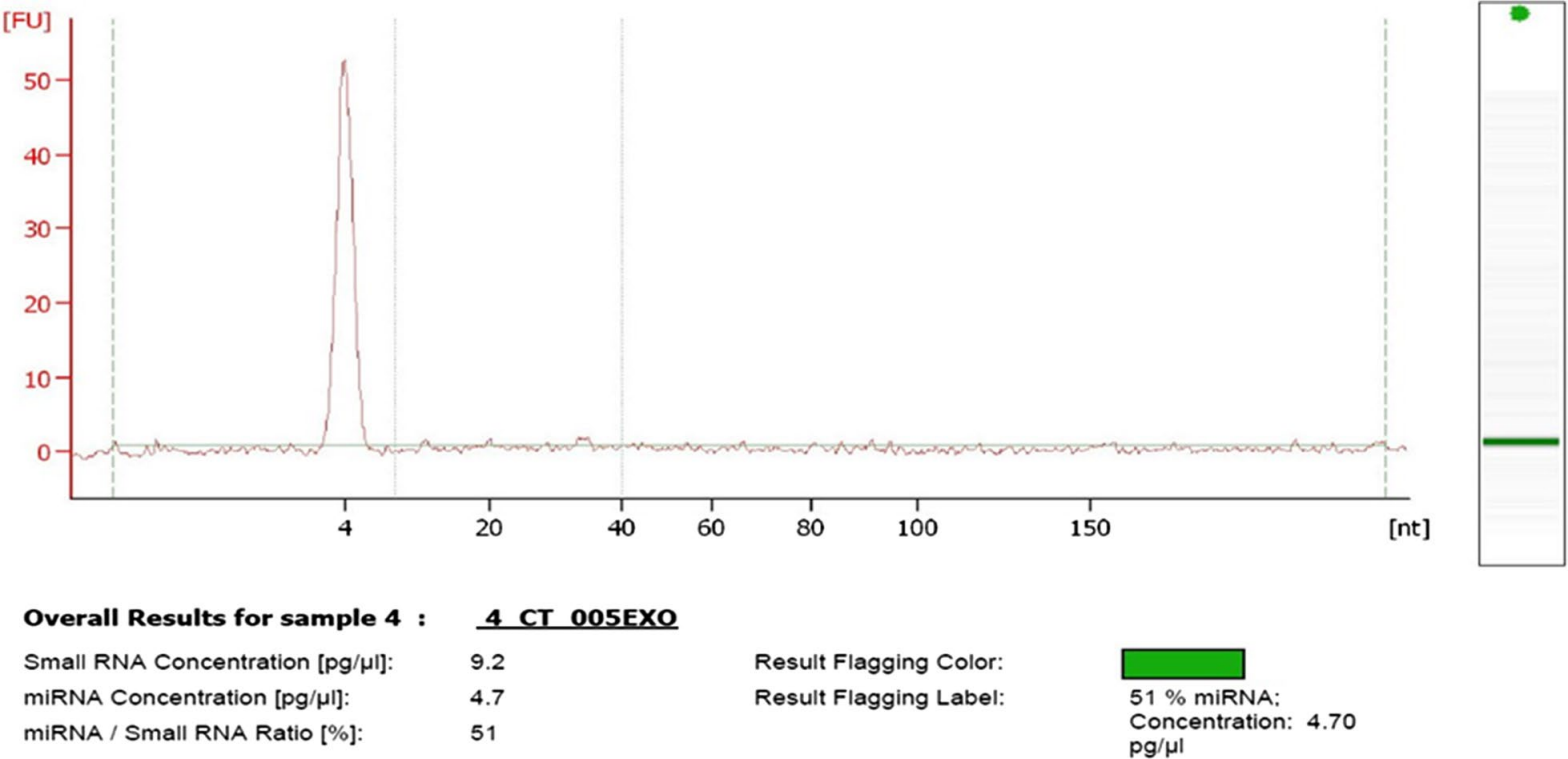
Small RNA profile from serum exosomes measured by Agilent Small RNA kit. The representative electropherograms showing nucleotide size (x-axis) between 4 and 40 as indicated my vertical lines is the region for miRNAs while peak at 4 nucleotide represents internal standards. The y-axis represents fluorescence units (FU)

Total Exosome RNA and Protein Isolation kit was used to extract RNA from exosome suspension following manufacturers instruction, which uses glass-fiber filters with affinity to RNA [26]. The miRNAs recovered from exosomes that were initially derived from 1 ml of serum was below 1000 pg/µl indicating low yield in both baseline and post-radiation samples, and controls. As an example, Agilent 2100 Bioanalyzer Small RNA Chip result showed RNA at concentration of 9.2 pg/µl while miRNA constituted approximately 51% of total RNA (Fig. 5). The qRT-PCR result based on average Ct values for all five miRNAs indicate successful recovery of miRNAs except for RNU48, from all the 25 samples. The data showed hsa-miR-21-5p (average Ct value = 27.82), and hsa-miR-7a-5p (average Ct value = 28.98) were in high copy numbers while hsa-miR-99b, hsa-miR-141-3p, hsa-miR-145 were less with average Ct values of 32.50, 34.72, 32.46 respectively.

**Fig. 6:**
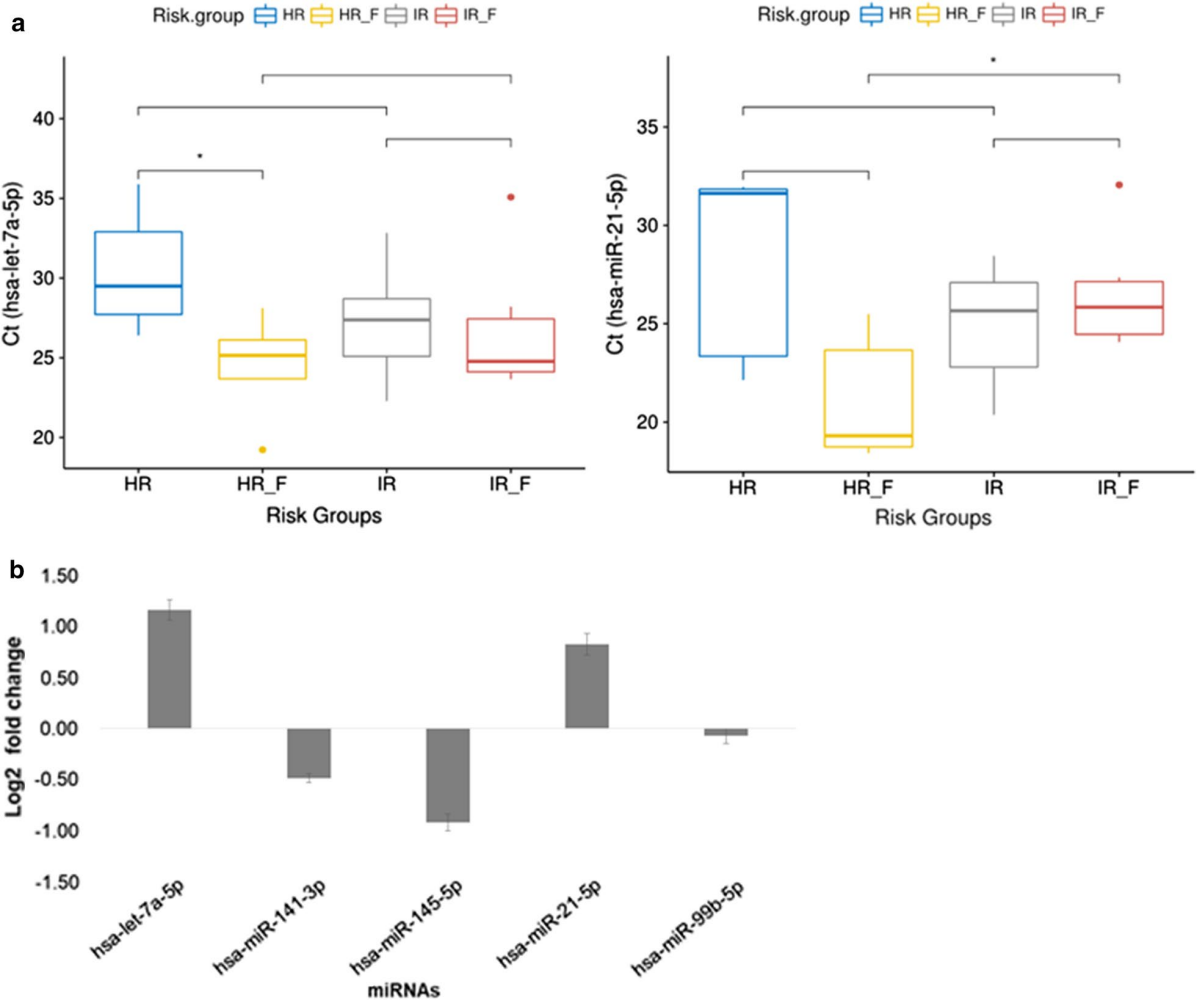
**a** Relative expression of hsa-let-7a-5p and hsa-miR-21-5p in risk groups. The relative expression of hsa-let-7a-5p, and hsa-miR-21-5p in risk groups were evaluated by measuring the difference in threshold cycle number. The expression of hsa-let-7a-5p was significantly different between HR, and HR_F (p = 0.037) while, hsa-miR-21-5p expression was significantly different between HR, and IR following radiation (p = 0.036). This may mean the function and role of hsa-miR-21-5p in PCa differs in response to radiation, based on risk category. *IR* intermediate risk without radiotherapy, *HR* high risk group without radiotherapy, *IR_F* intermediate risk post radiotherapy, *HR_F* high risk post radiotherapy. One asterisk indicates significant (p-value < 0.05) difference in miRNAs expression level between compared groups. **b** Fold change results for miRNAs. The fold change values of five miRNAs are shown. The y-axis represents log transformed 2^−ΔΔCt^ value calculated in radiation treated patients relative to pre-radiotherapy (baseline)

The comparison of all five miRNAs in risk groups categorized as before (IR, HR) and after radiotherapy (IR_F, HR_F) using Ct values gave mixed results; hsa-miR-141-3p, hsa-miR-145-5p, hsa-miR-99b-5p were not significantly different across risk groups. The evaluation of hsa-let-7a-5p, and hsa-miR-21-5p showed distinct expression in both risk groups (Fig. 6a). Especially, in high-risk group, both miRNA expression became more abundant post radiation which might indicate it has protective effect; the argument being the expression became comparable to control group without known prostate disorders. The expression of hsa-let-7a-5p, which could discriminate radiation response in HR group, was abundant in exosomes after radiation (p = 0.037). The expression of hsa-miR-21-5p, which could not discriminate HR from IR patients at baseline, was abundant in exosomes after radiation in HR groups only (*p *= 0.036). The miRNA expression referred to a reference group, baseline versus post-radiated samples showed heterogeneous distribution as expected (Fig. 6b). The upregulation of two miRNAs namely hsa-let-7a-5p (fold change 2.24) and hsa-miR-21-5p (fold change 1.77) potentially indicating an induction due to radiation.

## Discussion

Recent studies reported the variability in exosome subtypes and cargo composition emphasizing the importance of isolation and characterization methods. In clinical settings, the success of an optimal liquid biopsy workflow is dependent of small sample volumes that allow proper characterization of the exosomal cargo. A better characterization of exosomes origin through molecular and genetic analysis and changes induced by treatment could provide valuable knowledge for treatment personalization. Radiotherapy is a primary curative modality in PCa [27]. Although previous studies focused on isolation, and characterization of exosomes, variation in paired patient samples before and after radiation has never been investigated. To our knowledge, this is the first study to assess an exosome isolation workflow for PCa radiotherapy. We demonstrated that despite minimal volumes and extraction of exosomal miRNAs, qRT-PCR-based expression analysis is technically feasible.

Although a minimal volume threshold of serum required for optimum yield of exosomes is an important limiting factor, we have isolated exosomes from a volume of 1 ml. A titration study found that the volume of input serum positively correlates to the exosome yield [28]. In this study, median follow-up sampling time interval was 93 days (min 34; max 148) after radiation which is an important factor to be considered. From biological perspective, quantitative and qualitative variation of exosomes and its RNA molecules would be meaningful in defining their predictive markers for specific therapies, establishing a basis for detection of a temporal relationship [29, 30]. In general, higher concentration of exosomes in patient samples could indicate radiation specific induction; however, more specific molecules such as DNA damage markers deserve further investigation [14]. Moreover, exosome vesiculation is proposed to be influenced by multiple factors which may interfere with exosome release and its content [16]. The method used to isolate exosomes influences the quality and quantity of exosomes [28]. The PEG has long been used for precipitation of small particles such as viruses that may precipitate exosomes present in the samples (e.g. blood, cell culture supernatant) [31]. Despite low purity, enrichment of vesicles can be achieved by filtration and certain optimization methods that enriches vesicles significant in quality and quantity for biomarker research [18]. The input of less serum volumes may cause breaking of vesicles in other methods such as ultracentrifugation causing low particle recovery and biased downstream analysis [32]. The PEG reagent forms polymer for better precipitation of exosomes yet preserves their biological activities to be used in basic and clinical research [33]. In particular, PEG-based exosome isolation provides consistent measurements which are in general consensus to most extravesicular research findings [34].

The Nanosight measurements showed consistent data for exosomes that has been corroborated in other studies with conventional ultracentrifugation methods. This suggests that PEG method is a suitable tool in vesicles recovery from serum [35]. The Nanosight technology relies on laser light scattering microscopy on Brownian motion of the particles providing size-based particle count as well as respective concentration. This might have limitations in precisely capturing exosomes alone excluding noise created by lipoproteins, protein aggregates, and other biological vesicles from serum [19]. Another criticism of using NTA technology is the evidence of operator handling bias that may influence the accuracy and reproducibility of the measurements [36]. As the isolation and enrichment of exosomes from biofluids is an elaborate task, technical variations may impact interpretation of results, especially in comparing individual cases. Thus, sample grouping would arguably make more sense in the interpretation of radiation-induced changes. We have stratified our samples before and after irradiation. Patient variability may show endogenous variability in terms of size and distribution of exosomes, an ideal protocol in NTA that is applicable for each and every sample would be difficult to validate, despite certain possibilities to reduce those variation by optimizing NTA software settings [19, 37].

Our FACS experimental results are limited in outlining one possible approach in exosome characterization workflow, though implication of exosomal surface markers is briefly discussed. The FACS result may lead to a hypothesis that CD9 surface marker is less expressed compared to CD63 in serum exosomes from PCa patients. This may also indicate the exosomal sub-population theory regarding their concentration, heterogeneous surface markers, and contents are influenced by multiple factors (e.g. clinical phenotypes) [38]. In contrast, studies have also found exosomes representing higher amount of CD9 surface marker in advanced and chemo-resistant PCa compared to others [39]. Such variation showing selective enrichment of exosomes can also be due to methodological variation used for their isolation and processing [15, 40]. While NTA technologies are limited to size based discrimination, use of beads (4.5 µm diameter) labelled with surface markers for exosomes make it possible to characterize them by FACS [41]. In our pilot study, we used Dynabeads^®^ coated with primary monoclonal antibody specific for human exosomes surface antigen CD63 without knowing the concentration of optimal antibodies required for samples. A standard titration curve using standard exosome concentration will be essential to ensure most correct number of exosomes captured by beads. Heterogeneous exosome populations with varying levels of surface antigen (e.g. CD63, CD9) or their relative expression is important to consider which may allow to correctly interpret biological causes of (sub) population and concentration of exosomes in biofluids [42, 43]. It is important to note that multiple numbers of exosomes may bind on a bead which may potentially bias the exact number of exosomes in the given volume of specimen. The detection limit of flow cytometers is 200 nm which is the size threshold used to differentiate exosomes from other microvesicles [44]. Despite certain limitations by FACS, use of magnetic beads solve one important aspect of identifying exosome in biofluids [43]. Moreover, recent technical upgrades in FACS targeting multiple surface markers may provide desired level of standardization methods that can be widely used for routine exosomes characterization workflow in future [44, 45].

In subsequent RNA extraction step, other RNA species such as full length 18S and 28S rRNA peaks were not observed in electropherograms which is in congruence to other similar study [28]. The choice of exosome isolation method was found to selectively enrich miRNA expression during qRT-PCR e.g. miR-16, let-7a [46]. Due to low sample input volume, the yield of RNA was less and was difficult to measure by Qubit, Nanodrop [47]. The possible explanations could be due to multiple washing steps in protocol of Total Exosome RNA and Protein Isolation kit that washed away certain amounts of small RNA. The rate of miRNA synthesis at site of cancer and its half-life in biofluids determine their expression level. The turnover of miRNA in circulating exosome is not yet clear although the median half-life of other mRNA molecule is thought to be around 2 min [48]. Due to multiple reasons, it is challenging to confirm if the initial RNA volumes were comparable between samples, which would affect the downstream process till expression analysis [49]. A recent publication using mass spectrometry analysis showed small RNAs as major content of serum exosomes derived from colon cancer patients [50]. Novel approaches for scaling up exosome concentration may further enhance the possibility of recovering higher miRNA concentration in the serum.

In qRT-PCR analysis, there is no consensus in the choice of reference gene derived from exosomal RNA cargo for data normalization [51–53]. We tested RNU48 that has been used in other studies to normalize miRNAs in qRT-PCR analysis but gave “undetermined” result [54, 55]. RNU48 has been found to be dysregulated in certain cancer types such as breast and head and neck cancers that could introduce bias in the miRNA expression analysis [56]. Conclusively, the RNU48 was not present in detectable amount by qRT-PCR so we propose it should not be used as a reliable control for exosome study. We resorted in calculating average expression data for each set of samples for each miRNA target; a common approach used to normalization by taking mean Ct values from a set of miRNAs as similar to global mean normalization [57, 58].

The expression of hsa-let-7a-5p increased significantly post radiation in HR_F (p = 0.037). The hsa-miR-21-5p expression remained the same in the IR group while differed significantly in the HR_F (p = 0.036). The distinct expression of miRNAs in IR versus HR groups may have clinical ramifications (Fig. 6a). Four miRNAs (miRNA-21, miRNA-34a, miRNA-125, and miRNA-126) observed in comparison of PCa to benign prostatic hyperplasia demonstrate the heterogenity in miRNA expression [59]. With regard to the heterogeneity of expression data, another study found different sets of miRNAs (let-7c, let-7e, let-7i, miR-26a-5p, miR-26b-5p, miR-18b-5p and miR-25-3p) that discriminated benign prostatic hyperplasia patients from PCa [60]. While miR-21-5p better distinguished PCa from benign prostatic hyperplasia, exosomal let-7a-5p was found to be differentially expressed in patients with Gleason score ≥ 8 versus ≤ 6 [6]. The expression of hsa-let-7a family miRNAs is regulated in prostate cancer [61], and are altered by ionizing radiation [62]. Exosomal biogenesis still requires important elucidation, therefore its reliability as a disease biomarker depends on the study design, and also on the  specific clinical question [6, 63, 64]. Unless there is a stringent method to define association of exosome yield and its content with specific clinical endpoints, the cross-referencing from previous studies will still raise questions on its biomarker application [65]. With pilot samples, we demonstrated the feasibility to detect radiation-associated miRNAs in serum exosomes. Given limited sample size and important clinical heterogeneity, further studies in larger datasets are warranted.

## Conclusions

Our preliminary findings can be summarized as follows: (I) for exosome isolation, PEG-based assay is an efficient and reliable method, especially in clinical studies with limited sample volumes; (II) for exosome characterization, TEM, Nanosight and FACS can be used with reliable and reproducible results; (III) for miRNAs identification, the amplification was feasible using qRT-PCR; and (IV) differential expression of serum exosomal miRNAs are induced by PCa radiotherapy, which may have potential value as prognostic and predictive biomarkers.

### Limitations of the study

This publication is based on initial feasibility part of a larger clinical prospective study which has some limitations. Clinical and pre-clinical research on exosomes and its relation with cancer, specifically localized prostate cancer is still at early stage. Biomarker studies in localized PCa are challenging and require proper technical validation before implemention in larger sample cohort. To our knowledge, there has been little or no previous research done in localized disease comparing effects of radiotherapy in paired samples. The idea behind publishing early findings from small sample set was to confirm the feasibility of available techniques in exosome research. Likewise, miRNAs and its specificity to a definite disease state and treatment response might vary across individuals due to disease heterogeneity. Notably, in studies with smaller sample size, molecular and clinical heterogeneity of PCa may significantly affect miRNA expression [66]. Although ionizing radiation may induce release of exosomes at site of radiation, clinical presentation, size of tumor may influence expression and eventual release to biofluids distant from the site of radiation. There is paucity in data regarding comparative analysis on yield of exosomes at site of disease and distant biofluids. It is evident that PEG helps to isolate exosomes by forming a polymer complex, there is mixed interpretation on how residual PEG interacts with exosomes, and other particles present in serum. Similarly, methodological comparison of exosome isolation protocols has shown to include non-exosome associated miRNAs [67]. Our panel of miRNAs for targeted amplification is a unique set as it was selected from previously published PCa literature. Despite limitations, the proposed protocol is robust and reproducible which may be used as a standard workflow for exosome-based biomarker research (Additional file 2: Figure S1).

## Additional files

**Additional file 1: Table S1.** The functions and characteristics of miRNAs.

**Additional file 2: Figure S1.** Workflow of miRNA Profiling. The overall serum exosome isolation and its content characterization workflow consists primarily of two steps. (1) Isolation and enrichment of exosomes from serum. (2) miRNA extraction of exosomes and characterization by qRT-PCR.

## Abbreviations

Ago2: Argonaute2 protein complex
FACS: fluorescence-activated cell sorting
FITC: fluorescein isothiocyanate
FSC: forward scatter
HR: high risk
HR_F: high risk at follow-up
IR: intermediate risk
IR_F: intermediate risk at follow-up
miRNAs: microRNA
NGS: next generation sequencing
NTA: nanoparticle tracking analysis
PCa: prostate cancer
PE: phycoerythrin
PEG: polyethylene glycol
PEG: polyethylene glycol
PSA: prostate specific antigen
qRT-PCR: quantitative real time polymerase chain reaction
RT: radiation therapy
SAKK: Schweizerische Arbeitsgemeinschaft für Klinische Krebsforschung
SSC: side scatter
TEM: transmission electron microscopy
TNM: tumor node metastasis

## References

[1] Siegel R, Ma J, Zou Z, Jemal A (2014). Cancer statistics, 2014. CA Cancer J Clin.

[2] Murthy V, Rishi A, Gupta S, Kannan S, Mahantshetty U, Tongaonkar H, Bakshi G, Prabhash K, Bhanushali P, Shinde B (2016). Clinical impact of prostate specific antigen (PSA) inter-assay variability on management of prostate cancer. Clin Biochem.

[3] Di Meo A, Bartlett J, Cheng Y, Pasic MD, Yousef GM (2017). Liquid biopsy: a step forward towards precision medicine in urologic malignancies. Mol Cancer.

[4] Cocucci E, Racchetti G, Meldolesi J (2009). Shedding microvesicles: artefacts no more. Trends Cell Biol.

[5] Malla B, Zaugg K, Vassella E, Aebersold DM, Dal Pra A (2017). Exosomes and exosomal microRNAs in prostate cancer radiation therapy. Int J Radiat Oncol Biol Phys.

[6] Endzelins E, Berger A, Melne V, Bajo-Santos C, Sobolevska K, Abols A, Rodriguez M, Santare D, Rudnickiha A, Lietuvietis V (2017). Detection of circulating miRNAs: comparative analysis of extracellular vesicle-incorporated miRNAs and cell-free miRNAs in whole plasma of prostate cancer patients. BMC Cancer.

[7] Fabris L, Ceder Y, Chinnaiyan AM, Jenster GW, Sorensen KD, Tomlins S, Visakorpi T, Calin GA (2016). The potential of microRNAs as prostate cancer biomarkers. Eur Urol.

[8] Mendell JT, Olson EN (2012). MicroRNAs in stress signaling and human disease. Cell.

[9] Gandellini P, Rancati T, Valdagni R, Zaffaroni N (2014). miRNAs in tumor radiation response: bystanders or participants?. Trends Mol Med.

[10] Hatano K, Kumar B, Zhang Y, Coulter JB, Hedayati M, Mears B, Ni X, Kudrolli TA, Chowdhury WH, Rodriguez R (2015). A functional screen identifies miRNAs that inhibit DNA repair and sensitize prostate cancer cells to ionizing radiation. Nucleic Acids Res.

[11] Al-Mayah AH, Irons SL, Pink RC, Carter DR, Kadhim MA (2012). Possible role of exosomes containing RNA in mediating non targeted effect of ionizing radiation. Radiat Res.

[12] Suh SO, Chen Y, Zaman MS, Hirata H, Yamamura S, Shahryari V, Liu J, Tabatabai ZL, Kakar S, Deng G (2011). MicroRNA-145 is regulated by DNA methylation and p53 gene mutation in prostate cancer. Carcinogenesis.

[13] Fuse M, Nohata N, Kojima S, Sakamoto S, Chiyomaru T, Kawakami K, Enokida H, Nakagawa M, Naya Y, Ichikawa T, Seki N (2011). Restoration of miR-145 expression suppresses cell proliferation, migration and invasion in prostate cancer by targeting FSCN1. Int J Oncol.

[14] Dal Pra A, Locke JA, Borst G, Supiot S, Bristow RG (2016). Mechanistic insights into molecular targeting and combined modality therapy for aggressive, localized prostate cancer. Front Oncol.

[15] Lotvall J, Hill AF, Hochberg F, Buzas EI, Di Vizio D, Gardiner C, Gho YS, Kurochkin IV, Mathivanan S, Quesenberry P (2014). Minimal experimental requirements for definition of extracellular vesicles and their functions: a position statement from the International Society for Extracellular Vesicles. J Extracell Vesicles.

[16] Witwer KW, Buzas EI, Bemis LT, Bora A, Lasser C, Lotvall J, Nolte-’t Hoen EN, Piper MG, Sivaraman S, Skog J (2013). Standardization of sample collection, isolation and analysis methods in extracellular vesicle research. J Extracell Vesicles.

[17] Mohler J, Bahnson RR, Boston B, Busby JE, D’Amico A, Eastham JA, Enke CA, George D, Horwitz EM, Huben RP (2010). NCCN clinical practice guidelines in oncology: prostate cancer. J Natl Compr Cancer Netw.

[18] Rider MA, Hurwitz SN, Meckes DG (2016). ExtraPEG: a polyethylene glycol-based method for enrichment of extracellular vesicles. Sci Rep.

[19] Vestad B, Llorente A, Neurauter A, Phuyal S, Kierulf B, Kierulf P, Skotland T, Sandvig K, Haug KBF, Ovstebo R (2017). Size and concentration analyses of extracellular vesicles by nanoparticle tracking analysis: a variation study. J Extracell Vesicles.

[20] Lasser C, Eldh M, Lotvall J (2012). Size and concentration analyses of extracellular vesicles by nanoparticle tracking analysis: a variation study. J Vis Exp.

[21] van der Vlist EJ, Nolte-’t Hoen EN, Stoorvogel W, Arkesteijn GJ, Wauben MH (2012). Fluorescent labeling of nano-sized vesicles released by cells and subsequent quantitative and qualitative analysis by high-resolution flow cytometry. Nat Protoc.

[22] Kroh EM, Parkin RK, Mitchell PS, Tewari M (2010). Analysis of circulating microRNA biomarkers in plasma and serum using quantitative reverse transcription-PCR (qRT-PCR). Methods.

[23] Van Deun J, Mestdagh P, Sormunen R, Cocquyt V, Vermaelen K, Vandesompele J, Bracke M, De Wever O, Hendrix A (2014). The impact of disparate isolation methods for extracellular vesicles on downstream RNA profiling. J Extracell Vesicles.

[24] Lee K, Shao H, Weissleder R, Lee H (2015). Acoustic purification of extracellular microvesicles. ACS Nano.

[25] Li M, Rai AJ, DeCastro GJ, Zeringer E, Barta T, Magdaleno S, Setterquist R, Vlassov AV (2015). An optimized procedure for exosome isolation and analysis using serum samples: application to cancer biomarker discovery. Methods.

[26] Zeringer E, Li M, Barta T, Schageman J, Pedersen KW, Neurauter A, Magdaleno S, Setterquist R, Vlassov AV (2013). Methods for the extraction and RNA profiling of exosomes. World J Methodol.

[27] Dal Pra A, Souhami L (2016). Prostate cancer radiation therapy: a physician’s perspective. Phys Med.

[28] Helwa I, Cai J, Drewry MD, Zimmerman A, Dinkins MB, Khaled ML, Seremwe M, Dismuke WM, Bieberich E, Stamer WD (2017). A comparative study of serum exosome isolation using differential ultracentrifugation and three commercial reagents. PLoS ONE.

[29] Ouyang Y, Li D, Pater JL, Levine M (2005). The importance of temporal effects in evaluating the prognostic impact of joint ERPR expression in premenopausal women with node-positive breast cancer. Breast Cancer Res Treat.

[30] Tu H, Sun L, Dong X, Gong Y, Xu Q, Jing J, Long Q, Flanders WD, Bostick RM, Yuan Y (2015). Temporal changes in serum biomarkers and risk for progression of gastric precancerous lesions: a longitudinal study. Int J Cancer.

[31] Lewis GD, Metcalf TG (1988). Polyethylene glycol precipitation for recovery of pathogenic viruses, including hepatitis A virus and human rotavirus, from oyster, water, and sediment samples. Appl Environ Microbiol.

[32] Lobb RJ, Becker M, Wen SW, Wong CS, Wiegmans AP, Leimgruber A, Moller A (2015). Optimized exosome isolation protocol for cell culture supernatant and human plasma. J Extracell Vesicles.

[33] Niu Z, Pang RTK, Liu W, Li Q, Cheng R, Yeung WSB (2017). Polymer-based precipitation preserves biological activities of extracellular vesicles from an endometrial cell line. PLoS ONE.

[34] Tauro BJ, Greening DW, Mathias RA, Ji H, Mathivanan S, Scott AM, Simpson RJ (2012). Comparison of ultracentrifugation, density gradient separation, and immunoaffinity capture methods for isolating human colon cancer cell line LIM1863-derived exosomes. Methods.

[35] Tang YT, Huang YY, Zheng L, Qin SH, Xu XP, An TX, Xu Y, Wu YS, Hu XM, Ping BH, Wang Q (2017). Comparison of isolation methods of exosomes and exosomal RNA from cell culture medium and serum. Int J Mol Med.

[36] Gerritzen MJH, Martens DE, Wijffels RH, Stork M (2017). High throughput nanoparticle tracking analysis for monitoring outer membrane vesicle production. J Extracell Vesicles.

[37] Tong M, Brown OS, Stone PR, Cree LM, Chamley LW (2016). Flow speed alters the apparent size and concentration of particles measured using NanoSight nanoparticle tracking analysis. Placenta.

[38] Gould SJ, Raposo G (2013). As we wait: coping with an imperfect nomenclature for extracellular vesicles. J Extracell Vesicles.

[39] Mizutani K, Terazawa R, Kameyama K, Kato T, Horie K, Tsuchiya T, Seike K, Ehara H, Fujita Y, Kawakami K (2014). Isolation of prostate cancer-related exosomes. Anticancer Res.

[40] Yamashita T, Takahashi Y, Nishikawa M, Takakura Y (2016). Effect of exosome isolation methods on physicochemical properties of exosomes and clearance of exosomes from the blood circulation. Eur J Pharm Biopharm.

[41] Gardiner C, Ferreira YJ, Dragovic RA, Redman CW, Sargent IL (2013). Extracellular vesicle sizing and enumeration by nanoparticle tracking analysis. J Extracell Vesicles.

[42] Willms E, Johansson HJ, Mager I, Lee Y, Blomberg KE, Sadik M, Alaarg A, Smith CI, Lehtio J, El Andaloussi S (2016). Cells release subpopulations of exosomes with distinct molecular and biological properties. Sci Rep.

[43] Kowal J, Arras G, Colombo M, Jouve M, Morath JP, Primdal-Bengtson B, Dingli F, Loew D, Tkach M, Thery C (2016). Proteomic comparison defines novel markers to characterize heterogeneous populations of extracellular vesicle subtypes. Proc Natl Acad Sci USA.

[44] Pospichalova V, Svoboda J, Dave Z, Kotrbova A, Kaiser K, Klemova D, Ilkovics L, Hampl A, Crha I, Jandakova E (2015). Simplified protocol for flow cytometry analysis of fluorescently labeled exosomes and microvesicles using dedicated flow cytometer. J Extracell Vesicles.

[45] Nolte-’t Hoen EN, van der Vlist EJ, Aalberts M, Mertens HC, Bosch BJ, Bartelink W, Mastrobattista E, van Gaal EV, Stoorvogel W, Arkesteijn GJ, Wauben MH (2012). Quantitative and qualitative flow cytometric analysis of nanosized cell-derived membrane vesicles. Nanomedicine.

[46] Channavajjhala SK, Rossato M, Morandini F, Castagna A, Pizzolo F, Bazzoni F, Olivieri O (2014). Optimizing the purification and analysis of miRNAs from urinary exosomes. Clin Chem Lab Med.

[47] Garcia-Elias A, Alloza L, Puigdecanet E, Nonell L, Tajes M, Curado J, Enjuanes C, Diaz O, Bruguera J, Marti-Almor J (2017). Defining quantification methods and optimizing protocols for microarray hybridization of circulating microRNAs. Sci Rep.

[48] Baudrimont A, Voegeli S, Viloria EC, Stritt F, Lenon M, Wada T, Jaquet V, Becskei A (2017). Multiplexed gene control reveals rapid mRNA turnover. Sci Adv.

[49] Tiberio P, Callari M, Angeloni V, Daidone MG, Appierto V (2015). Challenges in using circulating miRNAs as cancer biomarkers. Biomed Res Int.

[50] Zhao L, Yu J, Wang J, Li H, Che J, Cao B (2017). Isolation and Identification of miRNAs in exosomes derived from serum of colon cancer patients. J Cancer.

[51] Vigneron N, Meryet-Figuiere M, Guttin A, Issartel JP, Lambert B, Briand M, Louis MH, Vernon M, Lebailly P, Lecluse Y (2016). Towards a new standardized method for circulating miRNAs profiling in clinical studies: interest of the exogenous normalization to improve miRNA signature accuracy. Mol Oncol.

[52] Blondal T, Jensby Nielsen S, Baker A, Andreasen D, Mouritzen P, Wrang Teilum M, Dahlsveen IK (2013). Assessing sample and miRNA profile quality in serum and plasma or other biofluids. Methods.

[53] Zeka F, Mestdagh P, Vandesompele J (2015). RT-qPCR-based quantification of small non-coding RNAs. Methods Mol Biol.

[54] Schwarzenbach H, da Silva AM, Calin G, Pantel K (2015). Data normalization strategies for microRNA quantification. Clin Chem.

[55] Torres A, Torres K, Wdowiak P, Paszkowski T, Maciejewski R (2013). Selection and validation of endogenous controls for microRNA expression studies in endometrioid endometrial cancer tissues. Gynecol Oncol.

[56] Gee HE, Buffa FM, Camps C, Ramachandran A, Leek R, Taylor M, Patil M, Sheldon H, Betts G, Homer J (2011). The small-nucleolar RNAs commonly used for microRNA normalisation correlate with tumour pathology and prognosis. Br J Cancer.

[57] Mestdagh P, Van Vlierberghe P, De Weer A, Muth D, Westermann F, Speleman F, Vandesompele J (2009). A novel and universal method for microRNA RT-qPCR data normalization. Genome Biol.

[58] Livak KJ, Schmittgen TD (2001). Analysis of relative gene expression data using real-time quantitative PCR and the 2(−Delta Delta C(T)) Method. Methods.

[59] Zedan AH, Blavnsfeldt SG, Hansen TF, Nielsen BS, Marcussen N, Pleckaitis M, Osther PJS, Sorensen FB (2017). Heterogeneity of miRNA expression in localized prostate cancer with clinicopathological correlations. PLoS ONE.

[60] Cochetti G, Poli G, Guelfi G, Boni A, Egidi MG, Mearini E (2016). Different levels of serum microRNAs in prostate cancer and benign prostatic hyperplasia: evaluation of potential diagnostic and prognostic role. Onco Targets Ther.

[61] Fredsoe J, Rasmussen AKI, Thomsen AR, Mouritzen P, Hoyer S, Borre M, Orntoft TF, Sorensen KD (2017). Diagnostic and prognostic microRNA biomarkers for prostate cancer in cell-free urine. Eur Urol Focus.

[62] Simone NL, Soule BP, Ly D, Saleh AD, Savage JE, Degraff W, Cook J, Harris CC, Gius D, Mitchell JB (2009). Ionizing radiation-induced oxidative stress alters miRNA expression. PLoS ONE.

[63] McDonald MK, Capasso KE, Ajit SK (2013). Purification and microRNA profiling of exosomes derived from blood and culture media. J Vis Exp.

[64] Nikolic I, Elsworth B, Dodson E, Wu SZ, Gould CM, Mestdagh P, Marshall GM, Horvath LG, Simpson KJ, Swarbrick A (2017). Discovering cancer vulnerabilities using high-throughput micro-RNA screening. Nucleic Acids Res.

[65] Pernot E, Hall J, Baatout S, Benotmane MA, Blanchardon E, Bouffler S, El Saghire H, Gomolka M, Guertler A, Harms-Ringdahl M (2012). Ionizing radiation biomarkers for potential use in epidemiological studies. Mutat Res.

[66] Boutros PC, Fraser M, Harding NJ, de Borja R, Trudel D, Lalonde E, Meng A, Hennings-Yeomans PH, McPherson A, Sabelnykova VY (2015). Spatial genomic heterogeneity within localized, multifocal prostate cancer. Nat Genet.

[67] Arroyo JD, Chevillet JR, Kroh EM, Ruf IK, Pritchard CC, Gibson DF, Mitchell PS, Bennett CF, Pogosova-Agadjanyan EL, Stirewalt DL (2011). Argonaute2 complexes carry a population of circulating microRNAs independent of vesicles in human plasma. Proc Natl Acad Sci USA.

