# Supplementary material for: Protocol for serum exosomal miRNAs analysis in prostate cancer patients treated with radiotherapy
Source: J Transl Med. 2018 Aug 13;16:223. doi: 10.1186/s12967-018-1592-6 (PMC6090775; doi:10.1186/s12967-018-1592-6)
Supplement: Supplementary file 1 — Additional file 1: Table S1. The functions and characteristics of miRNAs. [file 12967_2018_1592_MOESM1_ESM.doc]

Additional file 1: Table S1: The functions and characteristics of miRNAs

| Name | Accession No. | Functions | References |
| --- | --- | --- | --- |
| hsa-let-7a-5p | MIMAT0000062 | Negatively regulates RAS oncogene. | [1] |
| hsa-miR-141-3p | MIMAT0000432 | Tumor development, progression, and metastasis. | [2, 3] |
| hsa-miR-145-5p | MIMAT0000437 | Reduces efficiency of repair of radiation-induced .DNA double-strain breaks in prostate cell lines. | [4-6] |
| hsa-miR-21-5p | MIMAT0000076 | Regulates invasiveness through inhibiting translation of metalloproteinase inhibitor RECK, a regulator of cell invasion. | [7, 8] |
| hsa-miR-99b-5p | MIMAT0000689 | The mTOR induction contributes to irradiation resistance in (pancreatic) cancer. mir-99 family reduce rate of .DNA repair by both homologous recombination and non-homologous end joining | [9, 10] |

1. Johnson SM, Grosshans H, Shingara J, Byrom M, Jarvis R, Cheng A, Labourier E, Reinert KL, Brown D, Slack FJ: **RAS is regulated by the let-7 microRNA family.** *Cell* 2005, **120:**635-647.

2. Liu C, Liu R, Zhang D, Deng Q, Liu B, Chao HP, Rycaj K, Takata Y, Lin K, Lu Y, et al: **MicroRNA-141 suppresses prostate cancer stem cells and metastasis by targeting a cohort of pro-metastasis genes.** *Nat Commun* 2017, **8:**14270.

3. Lynch SM, O'Neill KM, McKenna MM, Walsh CP, McKenna DJ: **Regulation of miR-200c and miR-141 by Methylation in Prostate Cancer.** *Prostate* 2016, **76:**1146-1159.

4. Josson S, Sung SY, Lao K, Chung LW, Johnstone PA: **Radiation modulation of microRNA in prostate cancer cell lines.** *Prostate* 2008, **68:**1599-1606.

5. Gong P, Zhang T, He D, Hsieh JT: **MicroRNA-145 Modulates Tumor Sensitivity to Radiation in Prostate Cancer.** *Radiat Res* 2015, **184:**630-638.

6. Fuse M, Nohata N, Kojima S, Sakamoto S, Chiyomaru T, Kawakami K, Enokida H, Nakagawa M, Naya Y, Ichikawa T, Seki N: **Restoration of miR-145 expression suppresses cell proliferation, migration and invasion in prostate cancer by targeting FSCN1.** *Int J Oncol* 2011, **38:**1093-1101.

7. Bonci D, Coppola V, Patrizii M, Addario A, Cannistraci A, Francescangeli F, Pecci R, Muto G, Collura D, Bedini R, et al: **A microRNA code for prostate cancer metastasis.** *Oncogene* 2016, **35:**1180-1192.

8. Sapre N, Hong MK, Macintyre G, Lewis H, Kowalczyk A, Costello AJ, Corcoran NM, Hovens CM: **Curated microRNAs in urine and blood fail to validate as predictive biomarkers for high-risk prostate cancer.** *PLoS One* 2014, **9:**e91729.

9. Wei F, Liu Y, Guo Y, Xiang A, Wang G, Xue X, Lu Z: **miR-99b-targeted mTOR induction contributes to irradiation resistance in pancreatic cancer.** *Mol Cancer* 2013, **12:**81.

10. Mueller AC, Sun D, Dutta A: **The miR-99 family regulates the DNA damage response through its target SNF2H.** *Oncogene* 2013, **32:**1164-1172.
