# Supplementary figures and images for: Protocol for serum exosomal miRNAs analysis in prostate cancer patients treated with radiotherapy
Source: J Transl Med. 2018 Aug 13;16:223. doi: 10.1186/s12967-018-1592-6 (PMC6090775; doi:10.1186/s12967-018-1592-6)

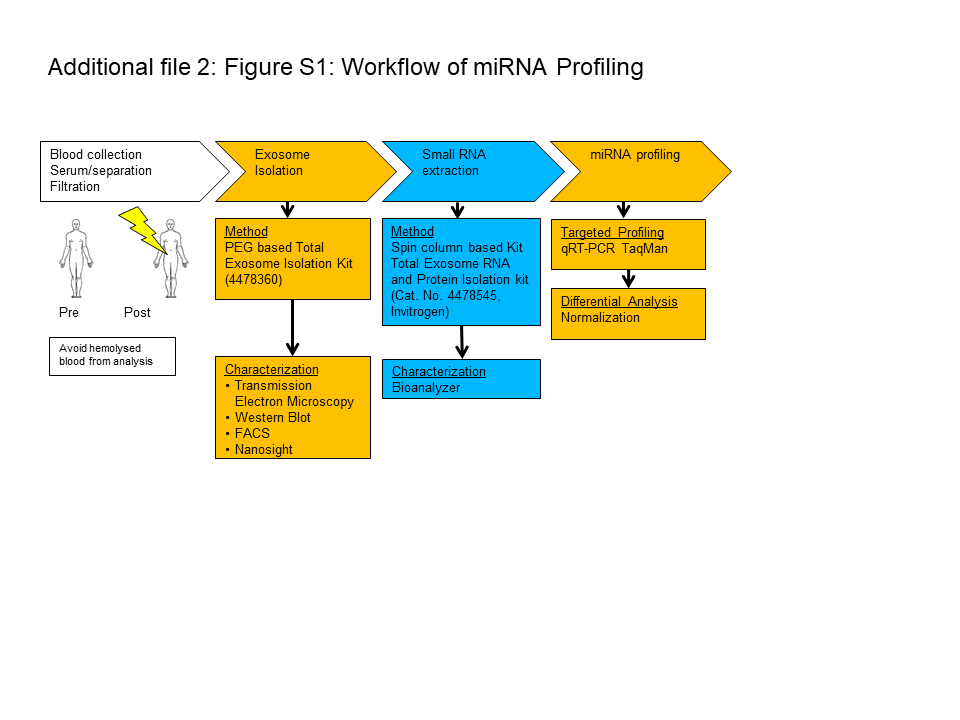

Supplement: Supplementary file 2 — Additional file 2: Figure S1. Workflow of miRNA Profiling. The overall serum exosome isolation and its content characterization workflow consists primarily of two steps. (1) Isolation and enrichment of exosomes from serum. (2) miRNA extraction of exosomes and characterization by qRT-PCR. [file 12967_2018_1592_MOESM2_ESM.tif]
